# From clinical trials to daily practice: how to adequately administer sulbactam-durlobactam? alone or combined with imipenem?

**DOI:** 10.1097/QCO.0000000000001148

**Published:** 2025-10-27

**Authors:** Cecilia Bonazzetti, Maddalena Giannella, Renato Pascale

**Affiliations:** a Infectious Diseases Unit, IRCCS Azienda Ospedaliero-Universitaria di Bologna; b Department of Medical and Surgical Sciences, University of Bologna, Bologna, Italy

**Keywords:** *Acinetobacter baumannii*, carbapenem-resistance, combination therapy, monotherapy, sulbactam-durlobactam

## Abstract

**Purpose of review:**

Sulbactam-durlobactam (SUL-DUR) is a novel β-lactam/β-lactamase inhibitor combination recently approved for carbapenem-resistant *Acinetobacter baumannii* (CRAB) infections. This review summarizes current knowledge on the optimal use of SUL-DUR, whether administered alone or in combination with carbapenems, particularly imipenem.

**Recent findings:**

Data from registrational trial demonstrate that SUL-DUR is an effective and well tolerated treatment option for CRAB severe infections. However, this trial assessed the efficacy of SUL-DUR exclusively in combination with imipenem. Real-world reports have described successful use of SUL-DUR in combination with carbapenems and other agents, particularly in complex or drug-resistant cases. Microbiological data suggest synergistic effects between SUL-DUR and carbapenems due to complementary inhibition of different penicillin-binding proteins.

**Summary:**

Combination therapy of SUL-DUR with carbapenems remains the preferred strategy in critically ill or high-risk patients. Future trials should specifically evaluate the comparative efficacy of monotherapy vs. combination regimens and establish which could be the best companion in the treatment of CRAB infections.

## INTRODUCTION

Over the past two decades, the global spread of carbapenem-resistant *Acinetobacter baumannii* (CRAB) has become a major public health concern, due to its association with poor clinical outcomes and limited treatment options [1]. According to the U.S. Centers for Disease Control and Prevention (CDC), CRAB is classified as an “urgent threat” pathogen, associated with over 8500 infections and approximately 700 deaths annually in the United States alone [2,3]. Furthermore, alarming resistance rates have been reported across Europe, with over 50% of *A. baumannii* isolates being carbapenem-resistant in at least 12 countries between 2012 and 2015 [1].

The Infectious Diseases Society of America (IDSA) recommends to administer sulbactam-based combination for the treatment of CRAB [4]. Ampicillin-sulbactam, the most widely available sulbactam-containing formulation, is the preferred agent for treating CRAB infections. It should be used as part of combination therapy and administered at high doses to achieve a total daily sulbactam component of 6–9 g. However, evidence from randomized clinical trials supporting the superior efficacy of ampicillin-sulbactam regimens compared to other treatment strategies remains limited [5]. Moreover, the absence of EUCAST breakpoints for sulbactam susceptibility in *Acinetobacter* species may further complicate its use in European centers [6]. Other therapeutic options include polymyxins (e.g., colistin), tigecycline, and minocycline, possibly in combination regimens to enhance efficacy [3,7]. However, these agents are frequently associated with suboptimal pharmacokinetic profiles, nephrotoxicity, or limited clinical efficacy, particularly in critically ill patients [8,9]. High expectations had been placed on cefiderocol, a siderophore cephalosporin with broad-spectrum antibacterial activity, including activity against CRAB. However, evidence from the registration trial showed less efficacy compared to traditional regimens (e.g., colistin) for the treatment of CRAB [10]. Nevertheless, real-world experiences have been much more promising [11–16]. Therefore, the IDSA guidance recommends the use of cefiderocol for CRAB infections that are refractory to other antibiotics or in cases where there is intolerance to other available agents, and it should be used as part of combination therapy [4].

This limited therapeutic armamentarium has led to treatment failures in many settings, prompting the need for new, targeted agents with reliable activity against CRAB [7,17]. In this context, sulbactam-durlobactam (SUL-DUR) has emerged as a promising new β-lactam/β-lactamase inhibitor combination specifically developed for the treatment of CRAB. Based on evidence from the registrational trial [18^▪▪^], the IDSA Guidance recommends SUL-DUR use in combination with imipenem-cilastatin or meropenem [4]. However, growing clinical reports support the effective use of SUL-DUR both as monotherapy and in combination with other agents.

The aim of this review is to summarize the current evidence on the optimal use of SUL-DUR, whether administered alone, with imipenem-cilastatin, or in combination with other antimicrobial agents. 

**Box 1:**
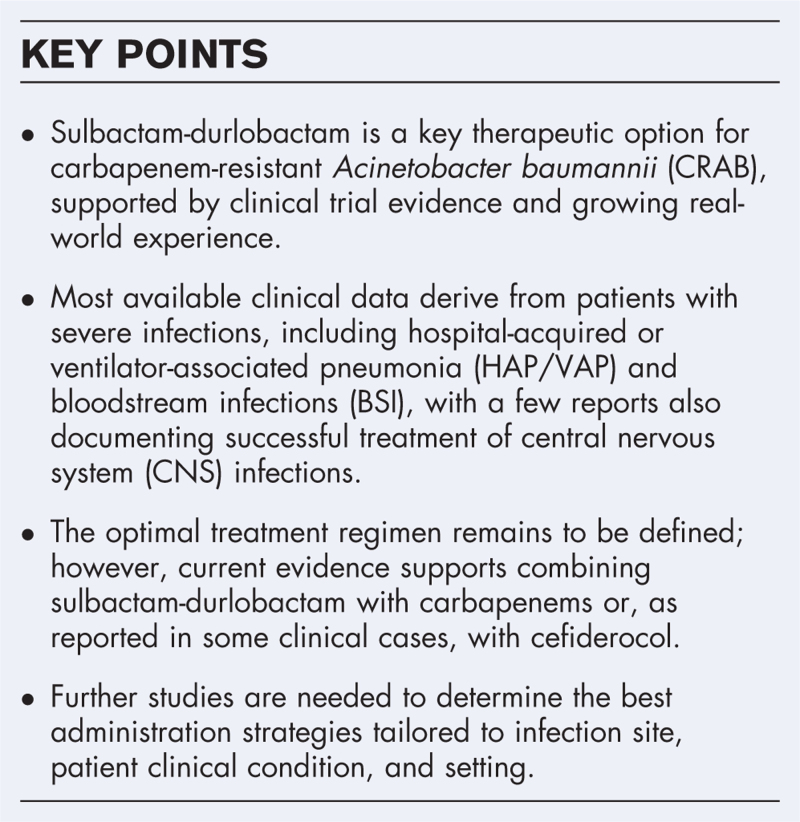
no caption available

## SULBACTAM-DURLOBACTAM

### General aspects

Sulbactam-durlobactam is a novel, pathogen-targeted β-lactam/β-lactamase inhibitor combination specifically designed to address infections caused by CRAB. SUL-DUR was approved by the U.S. Food and Drug Administration (FDA) in 2023 for hospital-acquired and ventilator-associated bacterial pneumonia (HABP/VABP) [19] due to *Acinetobacter baumannii–calcoaceticus* complex in adults. SUL-DUR has not yet been approved by the European Medicines Agency (EMA), and a pediatric investigation plan is currently underway [20].

Sulbactam, a penicillanic acid sulfone, is a β-lactamase inhibitor but has intrinsic antibacterial activity against *A. baumannii*, attributable to its affinity for penicillin-binding proteins PBP1 and PBP3, which are essential for bacterial cell wall synthesis [21]. However, its activity is significantly diminished in clinical isolates due to the expression of diverse β-lactamases, including Ambler class A TEM-1, class C ADC enzymes, and class D OXA-type carbapenemases [22].

Durlobactam is a next-generation diazabicyclooctane (DBO) non-β-lactam β-lactamase inhibitor. It restores the antimicrobial activity of sulbactam against *A. baumannii* by inhibiting several β-lactamases [23]. Indeed, durlbobactam has broad-spectrum inhibitory activity against Ambler class A, C, and D serine β-lactamases and is particularly potent against the OXA-type enzymes, which are key mediators of resistance in CRAB [23]. However, it does not inhibit class B metallo-β-lactamases (MBLs), although these are currently less common in *A. baumannii* populations [24^▪^]. Unlike earlier DBOs (e.g. avibactam), durlobactam binds more tightly to class D enzymes and exhibits greater stability, thereby restoring sulbactam's bactericidal activity [23]. Mechanistically, durlobactam operates via a reversible carbamoylation of the enzyme's active site, allowing it to detach intact and inhibit multiple enzymes, a property that enhances its catalytic efficiency [24^▪^].

### In-vitro activity of sulbactam-durlobactam

Multiple investigations have evaluated the *in-vitro* efficacy of SUL-DUR against clinical isolates of the *Acinetobacter baumannii–calcoaceticus* (ABC) complex, particularly those exhibiting carbapenem resistance. In an extensive international study, SUL-DUR *in-vitro* activity was tested in 5032 ABC isolates from different specimens collected from 33 countries between 2016 and 2021 [25^▪▪^] across Europe (42.1%), North America (29.9%), Asia/South Pacific (13.6%), Latin America (12.6%), and the Middle East (1.7%). Approximately 80% of the isolates analyzed were identified as *A. baumannii*, most derived from respiratory tract (54.3%), bloodstream (20.2%) and urinary tract infections (16.5%). When durlobactam was added to sulbactam at a fixed concentration of 4 μg/ml, the MIC_90_ was reduced dramatically dropping from 64 μg/ml (sulbactam alone) to 2 μg/ml for the combination [25^▪▪^]. Applying the FDA susceptibility breakpoint of ≤4 μg/ml [26], 98.3% of isolates were classified as susceptible to SUL-DUR. Moreover, the combination retained high efficacy across challenging resistance phenotypes, including carbapenem-resistant, colistin-resistant, and extensively drug-resistant (XDR) strains with over 96% of isolates in these subsets exhibiting MIC values within the susceptible range [25^▪▪^].

### Sulbactam-durlobactam resistance mechanisms

One of the most concerning features *of A. baumannii* is its extensive antimicrobial resistance profile. Carbapenem resistance is mainly driven by the production of OXA-type class D β-lactamases (e.g., OXA-23, OXA-24/40, OXA-58), often in combination with decreased outer membrane permeability and efflux pump overexpression [7,27]. Although SUL-DUR demonstrates potent *in vitro* activity against CRAB, resistance may still emerge through several mechanisms, mainly the expression of β-lactamases not inhibited by durlobactam or alterations in target sites:

(1) Metallo-β-lactamases (MBLs): as stated above, durlobactam is ineffective against class B MBLs, such as NDM-1, which are capable of hydrolyzing both sulbactam and other β-lactams despite the presence of durlobactam [24^▪^]. Even if currently rare in *A. baumannii*, MBL production represents a key limitation of SUL-DUR [25^▪▪^,^28^];
(2) Mutations in PBP3: point mutations near the active site serine of penicillin-binding protein 3 can reduce sulbactam binding affinity [28]. The most commonly identified mutations include T526S, A515V, and G523V, which alter the drug-target interaction and may lead to increased MICs, particularly when combined with β-lactamase production [28];
(3) Efflux pump activity: although not the primary driver, overexpression of efflux pumps (particularly AdeIJ efflux pump, due to mutations in *adeJ gene*) may contribute to increased MICs by reducing intracellular concentrations of the active compounds [29].

### Pharmacokinetics and pharmacodynamics

SUL-DUR demonstrates favorable pharmacokinetic/pharmacodynamic (PK/PD) properties, which support its use in critically ill populations [30–32]. Sulbactam and durlobactam are administered intravenously and show low to moderate protein binding (38% and 10%, respectively). Both agents have a relatively low volume of distribution, consistent with hydrophilic β-lactams [30].

Sulbactam's activity is primarily time-dependent, relying on the percentage of time that free drug concentrations remain above the minimum inhibitory concentration (%fT > MIC), with a 50% threshold associated with bactericidal activity. Conversely, durlobactam activity correlates to the ratio of the free area under the concentration-time curve to the MIC (fAUC/MIC). This parameter reflects the extent of β-lactamase inhibition over time, suggesting a mechanism of inhibition in which target occupancy of betalactamase increases progressively with drug exposure [32].

The approved dosing regimen of SUL-DUR consists of 1 g of sulbactam combined with 1 g of durlobactam administered every 6 h as a 3-h intravenous infusion. This schedule, which was employed in clinical trials, has been shown to achieve PK/PD targets across diverse populations, including in critically ill patients [19].

SUL-DUR is primarily cleared renally, requiring dose adjustments in patients with impaired renal function. Furthermore, patients with augmented renal clearance may require adjusted dosing intervals to maintain adequate drug exposure [32,33]. Importantly, both agents achieve therapeutic concentrations in epithelial lining fluid (ELF), with ELF-to-plasma AUC ratios of 50% for sulbactam and 36% for durlobactam [30,34].

## SULBACTAM-DURLOBACTAM CLINICAL EVIDENCE

### Registrational trial

The clinical development program for SUL-DUR followed a rational, stepwise approach from phase I to phase III trials (Table 1) to characterize its pharmacokinetics, safety, and efficacy.

**Table 1:** Clinical trial

First author	Country	Year of publication	Study phase	Time period	Study design	Population	Site of infection	Results/efficacy	Safety profile and adverse events
Rodvold KA *et al.* [33]	USA	2018	I	2017	Phase 1, multiple-dose open-label pharmacokinetic study to compare plasma, ELF, and alveolar macrophage concentrations	30 healthy adult subjects	N/A	Plasma exposure: Both agents displayed linear pharmacokinetics. Elimination half-lives were approximately 1.4 h (durlobactam) and 1.1 h (sulbactam). Pulmonary penetration: the ELF-to-unbound plasma AUC_0–6_ ratios were 0.41 for durlobactam and 0.81 for sulbactam, indicating good lung penetration. Drug concentrations in alveolar macrophages remained fairly constant throughout the 6-h dosing interval.	No serious adverse events reported. Mild or moderate adverse events occurred in 13.3% of participants: infusion site pain and minor lab abnormalities.
O’Donnell J *et al.* [35]	USA	2019	I	2017–2018	Phase 1, open-label, nonrandomized study.	34 adult subjects with varying degrees of renal function, including ESRD patients on HD.	N/A	Systemic exposure (Cmax and AUC) for both drugs increased in a predictable, linear fashion with declining renal function. CLr decreased proportionally with increasing impairment of both drugs. Approximately 33–41% of the dose was recovered in dialysis for ESRD patients. Hemodialysis effectively removed both agents from plasma, with dialysis clearance exceeding natural renal clearance in some cases.	No serious adverse events reported. Mild or moderate adverse events: dizziness, nausea.
Lickliter JD *et al.* [34]	Australia	2020	I	2016–2017	Randomized, placebo-controlled study Part A: single ascending doses of DUR (0.25 to 8 g) Part B: multiple ascending doses of DUR (0.25 to 2 g q6h) Parts C and D: DUR (1 g) with SUL (1 g) and/or IMI-CIL (0.5/0.5 g)	124 subjects (94 receiving DUR, 30 receiving placebo)	N/A	Renal excretion was a predominant clearance mechanism. No drug-drug interaction potential between DUR and SUL and/or IMI-CIL was identified. DUR safe and well tolerated administered either alone or in combination with SUL and/or IMI-CIL. DUR demonstrated linear dose-proportional exposure across the studied dose ranges.	No serious adverse events. Mild or moderate common adverse events: headache and catheter site phlebitis, nausea and somnolence.
Sagan O *et al.* [36]	20 sites: Belarus, Bulgaria, Russia, Ukraine	2020	II	2018	Double-blind, randomized, placebo-controlled study to evaluate the tolerability and PK of i.v. SUL-DUR administered with IMI in patients with cUTIs.	80 patients. 2 : 1 to receive either SUL-DUR (1 g/1 g q6h) or placebo, both with background IMI 500 mg q6h	Complicated UTI	Outcomes were comparable between groups: m-MITT success 76.6% (SUL-DUR) vs. 81.0% (placebo) Microbiological evaluable success: 80.0% vs. 81.0% Clinical success with SUL-DUR was 100% (3/3) vs. 75% (3/4) in the placebo arm.	Adverse events were mostly mild or moderate: headache (9.4%), diarrhea, nausea, and phlebitis (~3.8% each) Two patients in the SUL-DUR group discontinued due to adverse events (urticaria and elevated creatinine)
O’Donnell J *et al.* [32]	USA	2021	I	2020	Partially double-blind study conducted as a placebo-and active-controlled, single-infusion, three-way crossover study: - a single 3-h IV infusion of durlobactam 4 g (supratherapeutic dose); - a single 3-h IV infusion of placebo; - a single 3-h IV infusion of placebo plus an oral 400 mg dose of moxifloxacin (positive control).	32 healthy adult subjects	N/A	Durlobactam well tolerated with no serious adverse events.	The incidence of mild adverse events, such as fatigue, was similar between treatment arms.
Kaye KS *et al.* [18^▪▪^]	59 clinical sites in 16 countries	2023	III, phase A	2019–2021	Multicentre, randomised, active-controlled, phase 3, noninferiority clinical trial	Patients with infections caused by ABC (HABP/VAP or BSI) Sulb/durl or colistin both with imipenem/cilastatin N = 181 patients	HAP/VAP or BSI	28-day all-cause mortality: SUL-DUR: 19% vs. Colistin: 32%, a difference of –13.2% (95% CI –30.0 to 3.5). Nephrotoxicity was significantly lower with SUL-DUR than colistin (13% vs. 38%, *P* < 0.001).	Serious adverse events: 40% SUL-DUR group and 49% COL group. Treatment-related adverse events: 11% SUL-DUR 16% COL group.
			III, phase B		Multicentre open-label observational	Patients with infections caused by ABC not eligible for part A (colistin-R or intolerant) Sulb/durl with imipenem/cilastatin N = 28 patients	Mostly BSI	28-day all-cause mortality was 18%, clinical cure at TOC 71%, microbiological favorable assessment at TOC was 79%	

ABC, *Acinetobacter baumannii–calcoaceticus* complex; AUC, area under curve; CLr, renal clearance; Cmax, concentration maximum; COL, colistin; cUTI, complicated urinary tract infections; DUR, durlobactam; ELF, epithelial lining fluid; ESRD, end-stage renal disease; HD, hemodialysis; IMI, imipenem; IMI-CIL, imipenem-cilastatin; IV, intravenous; N/A, not available; PK, pharmacocynetics; SUL, sulbactam; TOC, timing of cure.

Phase I trials conducted in healthy volunteers established pharmacokinetic and safety profile of both sulbactam and durlobactam when administered alone or in combination with imipenem-cilastatin [20–22]. Studies by Rodvold [33] and Lickliter [34] showed that intravenous SUL-DUR administered as 1 g/1 g over 3 h every 6 h was well tolerated, with predictable plasma and ELF concentrations supportive of use in lower respiratory tract. O’Donnell [35] further demonstrated that both agents are primarily renally excreted and systemic exposure increases in a predictable manner with declining renal function, requiring dose adjustment in patients with moderate to severe renal impairment or end-stage renal disease. In a phase II study [36], SUL-DUR was evaluated in hospitalized adults with complicated urinary tract infections (cUTIs), including acute pyelonephritis. Patients received background therapy with imipenem-cilastatin and were randomized to either SUL-DUR or placebo. SUL-DUR was well tolerated, with adverse events primarily mild or moderate in severity. Although this trial was not powered for efficacy, microbiological success rates were similar between arms [36].

The pivotal phase III trial, known as the ATTACK study [18^▪▪^] was a randomized, noninferiority trial comparing SUL-DUR vs. colistin, both in combination with imipenem-cilastatin, for the treatment of adult patients with severe CRAB infections (e.g. Ventilator-Associated Pneumonia, VAP, Hospital-Acquired Pneumonia, HAP, Bloodstream Infection BSI). The trial was divided into two parts, according to CRAB susceptibility profiles (part A: colistin/polymyxin B susceptible strains; part B: colistin/polymyxin B resistant strains or where these agents were contraindicated due to nephrotoxicity or neuromuscular disorders). In part A, the study enrolled 181 patients, with 125 included in the microbiologically modified intention-to-treat population for efficacy analysis. The primary endpoint was 28-day all-cause mortality. SUL-DUR demonstrated noninferiority to colistin, with a mortality rate of 19% (12 out of 63) vs. 32.3% (20 out of 62), respectively, corresponding to a treatment difference of −13% (95% CI: −30 to 3.5). Notably, nephrotoxicity occurred significantly less frequently in the SUL-DUR arm compared to the colistin group (13% vs. 38%, *P* < 0.001), highlighting a major safety advantage.

Part B of the ATTACK trial, was designed as an open-label, nonrandomized observational cohort, aimed to evaluate the efficacy and safety of SUL-DUR in patients with infections caused by CRAB strains resistant to colistin/polymyxin B or where these agents were contraindicated. A total of 28 patients were enrolled in Part B, mainly with BSI. All patients received SUL-DUR plus imipenem-cilastatin, for 7–14 days, adjusted based on renal function. At day 28, all-cause mortality was 18% (5 out of 28), clinical cure at test-of-cure 71% (20 out of 28) and microbiological favorable response was 79% (22 out of 28). These outcomes were consistent with or better than those observed in the randomized controlled Part A, suggesting clinical benefit even in this high-risk population.

Notably, all patients in the ATTACK trial received combination therapy with imipenem–cilastatin, chosen for ensuring coverage in polymicrobial infections that occurred in almost 30% of cases. Indeed, monotherapy efficacy of SUL-DUR was not evaluated in this pivotal trial.

### Real-world experience

Emerging data are increasingly supporting the use of SUL-DUR, mainly as part of combination regimens, in complex or refractory real-world clinical settings (Table 2). Several published case reports have described the use of SUL-DUR via expanded access protocols for patients with XDR or PDR *A. baumannii* infections, particularly when all other therapeutic options had failed. These cases provide valuable insights into SUL-DUR application in clinical contexts beyond those included in RCTs.

**Table 2:** Real-life experience

First author	Country	Year	Type of study	Time period	Patients and site of infection	Microorganisms	Drugs	Timing	Efficacy	Adverse events
Zaidan N *et al.* [37]	USA	2021	Clinical Case	2021	55, F VAP	CRAB + *Pseudomonas aeruginosa*	SUL-DUR + FDC	14 days	Clinical improvement within 72 h of starting SUL-DUR (resolution of fever, reduction in respiratory secretions, hemodynamic stabilization).	No adverse events
Holger DJ *et al.* [39]	USA	2022	Clinical Case	2022	50, M VAP	CRAB	SUL-DUR + MPM	21 days	Empyema resolution after 21 days of antibiotic treatment.	No adverse events.
Tiseo G *et al.* [38]	Italy	2023	Clinical Case	2023	Female VAP	CRAB + *Pseudomonas aeruginosa*	SUL-DUR + COL	12 days	Clinical improved and microbiological eradication after 12 days of treatment.	No adverse events.
Tamma PD *et al.* [42^▪^]	USA	2024	Clinical Case	2023	42, F Meningitis	CRAB	SUL-DUR + MPM	14 days	Clinical resolution after 14 days of antibiotic treatment; microbiological eradication after 4 days of SUL-DUR (CSF cultures negative).	No adverse events.
Snowdin JW *et al.* [43]	USA	2024	Clinical Case	2023	75, F Postneurosurgical meningitis and empyema	CRAB	SUL-DUR + FDC + MIN	42 days	Clinical resolution after 6-week therapy.	No adverse events.
Kufel WD *et al.* [44]	USA	2025	Clinical Case	2023	59, M Bacteremic VAP	CRAB	SUL-DUR monotherapy	7 days	Microbiological recurrent after eleven days of SUL-DUR treatment.	No adverse events.
Webb A *et al.* [40]	USA	2025	Clinical Case	2024	71, F DDI (BSI) in KT	CRAB	SUL-DUR + FDC	14 days	Clinical cure after 9 days of antibiotics treatment.	No adverse events.
Mangioni D *et al.* [41]	Italy	2025	Clinical Case	2025	65, M Bacteremic VAP	CRAB	SUL-DUR + FDC	14 days	Clinical cure within 72 h after SUL-DUR start.	No adverse events.

BSI, bloodstream infection; COL, colistin; CRAB, carbapenem-resistant *Acinetobacter baumannii*; CSF, cerebro-spinal fluid; DDI, donor-derived infection; FDC, cefiderocol; KT, kidney transplant; MIN, minocycline; MPM, meropenem; PK/PD, pharmacocynetics/pharmacodynamics; SUL/DUR, sulbactam/durlobactam; VAP, ventilator-associated pneumonia.

Zaidan *et al.* [37] described a critically ill patient with COVID-19 developing VAP and septic shock caused by XDR *A. baumannii*. After failure of multiple agents, including high-dose ampicillin-sulbactam, polymyxin B, and eravacycline, SUL-DUR was administered in combination with cefiderocol. Clinical and microbiological cure were achieved within 72 h of starting combination therapy, with no adverse effects reported. The SUL-DUR MIC of *A. baumannii* strain was 4 mg/l, consistent with recommended susceptibility thresholds.

Tiseo *et al.* [38] described another case of a severely burned patient with VAP due to *CRAB* resistant to both colistin and cefiderocol. The patient was treated with SUL-DUR in combination with colistin due to concomitant carbapenem-resistant *Pseudomonas aeruginosa* isolation from respiratory samples. The CRAB isolate exhibited a SUL-DUR MIC of 1.5 mg/l, and whole genome sequencing revealed multiple resistance determinants (e.g., blaOXA-23, blaOXA-66, PBP3 mutations). The patient achieved microbiological eradication and clinical improvement. Holger *et al.* [39] reported the use of SUL-DUR in combination with meropenem in a patient with necrotizing pneumonia and empyema caused by XDR *A. baumannii*. This case was notable for SUL-DUR MIC of 8 mg/l, just above preliminary breakpoints. However, the addition of meropenem resulted in a synergistic effect, reducing the MIC to 4 mg/l at *in vitro* testing. Webb *et al.* [40] described a kidney transplant recipient who acquired donor-derived CRAB BSI. The patient was initially treated with SUL-DUR plus cefiderocol, achieving clinical and microbiological success. The therapy was later de-escalated to cefiderocol monotherapy after resolution of the acute phase. The combination was selected based on susceptibility data and to avoid nephrotoxicity in a high-risk patient.

Recently, Mangioni *et al.* [41], reported a case of a 65-year-old man with septic shock caused by bacteremic pneumonia due to CRAB. The patient was initially treated with a complex combination regimen including cefiderocol, ampicillin/sulbactam, amikacin, and tigecycline, without clinical improvement. Therefore, SUL-DUR was requested as an imported medication from the USA and administered in combination with cefiderocol. Whole-genome sequencing of blood and respiratory samples identified two distinct CRAB strains with different SUL-DUR MIC values (4 mg/l and 0.5 mg/l, respectively). The combination therapy led to a rapid clinical improvement. This case highlights the potential benefit of a combination therapy regimens in treating severe CRAB infections, particularly when MIC values are near the susceptibility breakpoint.

As for difficult to treat infection sites, two case reports reported the use of SUL-DUR for central nervous system infections. Tamma *et al.* [42^▪^] described the first case of CRAB meningitis successfully treated with SUL-DUR in combination with meropenem, following failure of cefiderocol and high-dose ampicillin–sulbactam. Drug concentrations of SUL-DUR in CSF were adequate despite augmented renal clearance, and the patient achieved sustained microbiological cure. Snowdin *et al.* [43] reported a case of postneurosurgical CRAB meningitis and empyema, successfully managed with a combination regimen of SUL-DUR, cefiderocol and minocycline for six weeks. Microbiological cure and full recovery were achieved. In vitro synergy testing supported the use of this triple combination.

Finally, regarding data for SUL-DUR monotherapy, in a PK/PD assessment of SUL-DUR Kufel [44] evaluated its use as monotherapy in a patient on continuous venovenous hemofiltration (CVVH) with bacteremic VAP due to a CRAB strain showing a SUL-DUR MIC of 4 mg/l. Initial microbiological response was achieved, but relapse occurred, and the patient ultimately died.

## CONCLUSION

According with reported data from clinical trials and real-world experiences, the optimal administration strategy of SUL-DUR remains to be established.

A growing microbiological and structural evidences supports the synergistic effect between SUL-DUR and carbapenems, especially imipenem. Veeraraghavan *et al.* [45] demonstrated that the combination of sulbactam, durlobactam, and imipenem resulted in significant reductions in MIC and exhibited bactericidal activity against CRAB strains harbouring class D β-lactamases. These findings are explained by sulbactam ability to target PBP1 and PBP3, while imipenem provides complementary inhibition of PBP2, together leading to more effective inhibition of bacterial cell wall synthesis [45]. Therefore, the combination of sulbactam, durlobactam, and imipenem may function as a strategy with additive or synergistic effect, rather than SUL-DUR merely being “supported” by a carbapenem [45,46]. These findings suggest that the clinical efficacy of SUL-DUR may be potentiated by co-administration with carbapenem, supporting a combination strategy in serious infections where multiple PBPs must be inhibited for optimal bacterial killing [1,45].

It is worth mentioning that in clinical practice SUL-DUR was also successfully combined with cefiderocol [37,40,41,43], this usually shows *in vitro* activity against CRAB. Thus, in our opinion, the best companion to SUL-DUR for the treatment of CRAB infections should be yet investigated.

Another critical factor to use SUL-DUR as a part of combination regimen, is the frequent occurrence of polymicrobial infections in critically ill patients. Currently, there are few data supporting the efficacy of SUL-DUR against pathogens other than *A. baumannii*. Thus, empiric combination therapy remains mandatory in polymicrobial infections, especially in the early phase of treatment [3,9].

A potential role for SUL-DUR monotherapy [44] could be hypothesized in selected patient populations with confirmed monomicrobial CRAB infections, lower MIC values, and no evidence of coinfection. However, it should be noted that all clinical trials assessing SUL-DUR's efficacy evaluated this drug within a combination regimen with imipenem.

Therefore, until further data are available, combination therapy remains the standard of care, especially in critically ill or immunocompromised patients [47].

## Acknowledgements

*None.*

### Financial support and sponsorship

*None.*

### Conflicts of interest

*There are no conflicts of interest.*

## REFERENCES AND RECOMMENDED READING

Papers of particular interest, published within the annual period of review, have been highlighted as:

▪ of special interest
▪▪ of outstanding interest
